# Targeted Inhibition of FTO Demethylase Protects Mice Against LPS-Induced Septic Shock by Suppressing NLRP3 Inflammasome

**DOI:** 10.3389/fimmu.2021.663295

**Published:** 2021-05-04

**Authors:** Jiahui Luo, Faxi Wang, Fei Sun, Tiantian Yue, Qing Zhou, Chunliang Yang, Shanjie Rong, Ping Yang, Fei Xiong, Qilin Yu, Shu Zhang, Cong-Yi Wang, Jinxiu Li

**Affiliations:** ^1^ The Center for Biomedical Research, NHC Key Laboratory of Respiratory Diseases, Department of Respiratory and Critical Care Medicine, Tongji Hospital, Tongji Medical College, Huazhong University of Science and Technology, Wuhan, China; ^2^ Department of Critical Care Medicine, The Second Xiangya Hospital, Central South University, Changsha, China

**Keywords:** FTO, *N*^6^-methyladenosine, entacapone, inflammasome, sepsis

## Abstract

Sepsis refers to the systemic inflammatory response syndrome caused by infection. It is a major clinical problem and cause of death for patients in intensive care units worldwide. The Fat mass and obesity-related protein (FTO) is the primary *N* ^6^-methyladenosine demethylase. However, the role of FTO in the pathogenesis of inflammatory diseases remains unclear. We herein show that nanoparticle-mediated *Fto*-siRNA delivery or FTO inhibitor entacapone administration dramatically inhibited macrophage activation, reduced the tissue damage and improved survival in a mouse model of LPS-induced endotoxic shock. Importantly, ablation of FTO could inhibit NLRP3 inflammasome through FoxO1/NF-κB signaling in macrophages. In conclusion, FTO is involved in inflammatory response of LPS-induced septic shock and inhibition of FTO is promising for the treatment of septic shock.

## Introduction

Recently, there are more than 18 million cases of severe sepsis worldwide each year. This disease refers to systemic inflammatory response syndrome caused by infections (1, 2). Infectious factors in sepsis activate the mononuclear macrophage system and other inflammatory response cells, resulting in the production and release of multiple inflammatory mediators. Sepsis is a major clinical problem and the leading cause of death in patients in intensive care units worldwide (3, 4). Thus, the development of novel effective treatments for sepsis is urgently needed.

NLRP3 inflammasome, causing the maturation and secretion of interleukin-1β (IL-1β), plays a critical role in the inflammatory response (5, 6). Upon normal circumstances, the active NLRP3 inflammasome helps to recruit immune cells to the site of infection and trigger the adaptive immune response (7). However, under pathological conditions, the aberrant activation of NLRP3 inflammasome can lead to the occurrence of inflammatory diseases, including septic shock. Numerous studies have shown that NLRP3 inflammasomes are associated with lipopolysaccharide (LPS)-induced septic shock (8–10). The level of IL-1β in plasma is associated with the severity of shock (11). In addition, targeting NLRP3 inflammasomes through gene editing can alleviate the acute inflammation of LPS-induced endotoxin shock (12). Considering the important role of inflammasome in the pathogenesis of sepsis, strategies aimed at regulating its activation may be beneficial for sepsis treatment.

*N* ^6^-methyladenosine (m^6^A) is the most abundant internal modification of messenger RNA (mRNA) and non-coding RNA in eukaryotic cells (13–15). The Fat mass and obesity-related protein (FTO) belongs to the family of Fe^2+^ and α-ketoglutarate-dependent oxygenase, which mainly catalyze the m^6^A demethylation (16). It is involved in multiple mRNA-related processes, including transcriptional stability, alternative splicing, mRNA translocation and protein translation (17–21). In the recent years, FTO has been widely regarded as an attractive biological target owing to its function on the mRNA modification. Targeted inhibition of FTO has been found to reduce body weight and regulate liver gluconeogenesis in diet-induced obese mice (22). However, it is unclear whether targeting FTO can be used to treat inflammatory diseases, especially for septic shock. Entacapone was previously considered to be a catechol-O-methyltransferase (COMT) inhibitor for the treatment of Parkinson’s disease (23). It has recently been identified as an effective chemical inhibitor of FTO. Structural and biochemical studies demonstrated that entacapone can directly bind to FTO and inhibit the demethylation activity (22). Therefore, entacapone was used as FTO inhibitor for treatment in this study.

In the recent years, FoxO1 has been confirmed to participate in regulating the production of IL-1β by macrophages, suggesting that FoxO1 signaling through NF-κB participates in pro-inflammatory cytokine production (24). Moreover, it has been found that FoxO1 can mediate the activation of NLRP3 inflammasome. Inhibition of FoxO1 by using of the molecule inhibitors could block NLRP3 inflammasome assembly and activation (25). Thus, we hypothesize that FTO is involved in LPS-induced septic shock and targeted inhibition of FTO demethylase might protect mice against LPS-induced septic shock by suppressing NLRP3 inflammasome *via* FoxO1/NF-κB signaling.

## Materials and Methods

### Human Samples

Twenty-four healthy volunteers and 15 septic patients were enrolled and classified according to the criteria of the Third International Consensus Definitions for Sepsis and Septic Shock (Sepsis-3) (1). Peripheral blood samples were collected after receiving a written informed consent from septic patients. The experiment was approved by the Ethics Committee of Tongji Medical College of Huazhong University of Science and Technology.

### Reagents

LPS and nigericin were purchased from Sigma-Aldrich (St. Louis, MO). NF-kB inhibitor, QNZ (EVP4593), was supplied by MedChemExpress (New Jersey, USA). Antibodies against FTO, NLRP3, FoxO1, P65, p-P65, IL-1β and Cleaved-IL-1β (Asp117) were obtained from Cell Signaling Technologies (Beverly, MA). ELISA kits of IL-1β, interleukin-6 (IL-6), interferon-γ (IFN-γ), tumor necrosis factor-α (TNF-α), interleukin-10 (IL-10) and interleukin-12(p70) (IL-12(p70)) were purchased from eBioscience (San Diego, CA). Brilliant Violet 421™ anti-mouse F4/80 antibody, PE anti-mouse/human CD11b antibody, FITC anti-mouse I-A/I-E antibody, APC anti-mouse CD80 antibody, PE/Cy7 anti-mouse CD86 antibody, FITC anti-mouse Ly-6G antibody and APC anti-mouse CD40 antibody were obtained from BioLegend (San Diego, CA, USA). Lipidoid (C12-200) was supplied by Xinjiahecheng Medical Chemistry Corporation (Wuhan, Hubei, China). mPEG2000-DEG was purchased from NOF Corporation (Tokyo, Japan).

### Mice

C57BL/6 mice (6–8 weeks old) were purchased from the Jackson’s Laboratory (Bar Harbor, ME, USA) and maintained in a specific pathogen free facility. Male mice were used in all animal studies. All experimental mice were housed individually in ventilated cages in a pathogen-free facility with a 12 h light/dark cycle and were fed with a standard mouse chow diet. siRNA-loaded liposomes were injected into mice 2 days before LPS challenge (15 mg/kg). Survival rate of mice were monitored. Other groups of mice were injected with 10 mg/kg entacapone before the systemic injection of LPS. All procedures involving animals were approved by the Tongji Hospital Animal Care and Use Committee in accordance with the National Institutes of Health guidelines.

### Cell Culture

Murine bone marrow derived macrophages (BMDMs) were differentiated with macrophage colony stimulating factor as previously reported (26). Briefly, the mouse bone marrow cells were flushed out from the femur and tibia with a syringe. Then the cells were filtered through a 70 µm filter. The red blood cells were lysed. Then, the cells were cultured with a medium containing macrophage colony stimulating factors. Finally, the differentiated BMDMs were treated with the indicated stimulation, and collected for quantitative RT-PCR and Western blot analyses.

### Activation of NLRP3 Inflammasome

First, the indicated concentration of entacapone and the same amount of DMSO were added to the cell culture medium. Then stimulate the BMDMs with 1 μg/mL LPS for 5 h. Nigericin (20 μM) was added to the cell culture medium for 30 min to induce inflammasome activation.

### siRNA Transfection

The siRNA against *Fto* was purchased from RiboBio Co., Ltd (Guangzhou, P. R. China). The sequences for the *Fto* siRNA are as follows: sense strand 5′-GGCAGAGATCCTGATACTT-3′. Then, Lipofectamine 3000 reagent (Invitrogen, Carlsbad, CA, USA) was used to perform siRNA transfection as previously described (27). A *scramble* siRNA duplex served as the negative control.

### *In Vivo* Biodistribution of the Liposomes

DiI-loaded liposomes were prepared as previously described (28). The mice were intraperitoneally injected with liposomes and anesthetized at different time points. The peritoneal fluid was collected for fluorescence analysis.

### Preparation and Characterization of siRNA-Loaded Liposomes

siRNA-loaded liposomes were prepared as described previously (29, 30). Briefly, lipoid, cholesterol, DSPC, and mPEG-DMG were dissolved in ethanol at a specific molar ratio. At the same time, siRNA was dissolved in citrated buffer (10 mM, pH 3). Then, the liposomes and siRNA were mixed rapidly by vortex.

### Western Blot Analysis

Western blot analysis was conducted by using established techniques (27, 31). Briefly, the cells were lysed on ice with RIPA lysis buffer (Biyuntian, Shanghai, China). Then, the Western blot analysis was performed using indicated primary antibodies. β-actin served as a loading control.

### Quantitative RT-PCR Analysis

Total RNA was isolated from human monocytes or murine BMDMs using the Trizol™ reagent (Takara, Japan). Real-time PCR was performed using the SYBR Green PCR master mix (Applied Biosystems, South San Francisco, CA, USA) in the ABI Prism 7500 Sequence Detection System (Applied Biosystems, South San Francisco, USA). The following primers were used: human *IL-1β* forward, 5′-CCACAGACCTTCCAGGAGAATG-3′ and reverse, 5′- GTGCAGTTCAGTGATCGTACAGG-3′; human *FTO* forward, 5′-ACTTGGCTCCCTTATCTGACC-3′ and reverse, 5′-TGTGCAGTGTGAGAAAGG CTT-3′; human *18S* forward, 5′-GTAACCCGTTGAACCCCATT-3′ and reverse, 5′*-*CCATCCAATCGGTAGTAGCG-3′; mouse *IL-1β* forward, 5′-GGATGAGGACAT GAGCACCT-3′ and reverse, 5′-GGAGCCTGTAGTGCAGTTGT-3′; mouse *Fto* forward, 5′-TCACAGCCTCGGTTTAGTTC-3′ and reverse, 5′- GCAGGATCAAAGGATTTCAACG-3′; and mouse *β-actin* forward, 5′-AGCCATGTA CGTAGCCATCC-3′ and reverse, 5′- CTCCAGCTGTGGTGGTGAA-3′. The relative RNA amount was normalized with *18S* or *β-actin* RNA.

### Quantification of Total m^6^A Level

Total mRNA m^6^A levels were detected by EpiQuik™ m^6^A RNA Methylation Quantification Kit (Epigentek). Total RNA was isolated from human monocytes or murine BMDMs using the Trizol™ reagent (Takara, Japan) and the concentration was detected using a NanoDrop spectrophotometer 2000 (Thermo Fisher Scientific). Measurements were performed to the manufacturer’s instructions by using colorimetric ELISA assays. The input RNA amount was 200 ng per reaction. m^6^A% was calculated to quantify the relative m^6^A RNA methylation levels of different RNA samples.

### Terminal Deoxynucleotidyl Transferase dUTP Nick End Labeling Assay

The terminal deoxynucleotidyl transferase dUTP nick end labeling assay (TUNEL) was carried out using a One-Step TUNEL Apoptosis Assay Kit (Biyuntian, Shanghai, China). The slides were treated with 20 μg/mL DNase-free protease K for 20 min at room temperature. Then, the slides were washed with PBS. The TUNEL reaction mixture was added to the sample and incubated for 60 min at 37 °C. DAPI was used to stain nuclei simultaneously. The TUNEL-positive cells were detected under a fluorescence microscope.

### Flow Cytometry Analysis

The mice were euthanized and the cells were obtained from the peritoneum and spleens. The flow cytometric analysis was performed using fluorophore-conjugated antibodies as previously described (32). Data were analyzed using FlowJo V10 software.

### Statistical Analysis

The Kaplan-Meier method was used for survival analysis. Other results were expressed as the mean ± SEM, and comparisons were accomplished by the Student’s *t* test or ANOVA as appropriate. In all cases, *P* < 0.05 was considered statistically significant. All *in vitro* studies were conducted with at least three replications. Statistical analyses of the data were conducted using the GraphPad Prism 7.00 software (GraphPad Software Inc., San Diego, CA).

## Results

### FTO Expression Is Correlated With IL-1β Expression in Peripheral Blood Monocytes of Septic Shock Patients

**Figure 1:**
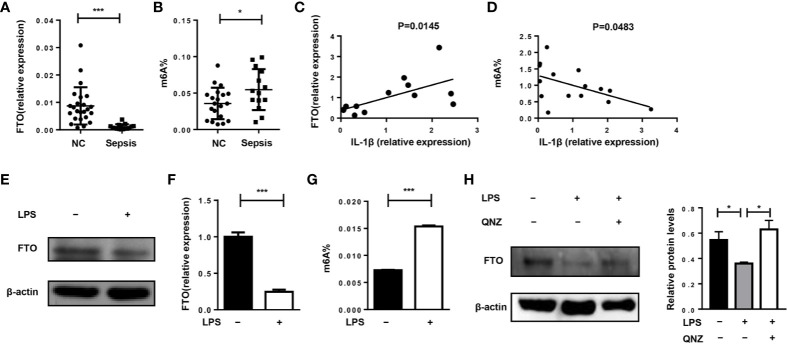
FTO expression is correlated with IL-1β expression in monocytes of septic shock patients. **(A, B)** qPCR analysis of expression of FTO and the m^6^A level in peripheral monocytes of septic patients (n = 15) and healthy volunteers (n = 24). **(C)** Correlation between FTO gene expression (n = 12) and m^6^A level (n = 15) **(D)** in peripheral monocytes from human patients. Murine BMDMs were treated or untreated with 500 ng/ml LPS. FTO expression was analyzed by western blot **(E)** and qPCR **(F)**. **(G)**The m^6^A level was measured by colorimetric ELISA assay. **(H)** LPS-stimulated BMDMs were pretreated with or without 5 μM QNZ(EVP4593) prior to the LPS treatment. FTO expression was measured by western blot. β-actin was used as the loading control. Data are shown as mean ± SEM; *P < 0.05; ***P < 0.001 (two-tailed unpaired t-test). Pearson’s correlation analysis was performed in **(C, D)**.

First, we want to investigate whether FTO expression and m^6^A level are related to the pathogenesis of sepsis in humans. Compared with healthy volunteers, FTO expression in monocytes of patients with sepsis was significantly reduced (**Figure 1A**). The m^6^A level in septic patients was elevated (**Figure 1B**). Interestingly, FTO expression was correlated with higher IL-1β expression in monocytes of septic patients (**Figure 1C**). The m^6^A level was significantly lower correlated with higher IL-1β expression (**Figure 1D**). In addition, LPS stimulation can decrease the expression of FTO in murine primary macrophages with significantly higher m^6^A level (**Figures 1E–G**). However, the FTO expression was increased after QNZ, the NF-kB inhibitor, treatment (**Figure 1H**).

We hypothesized that the lower FTO expression level would attenuate the disease progression but FTO expression might be inhibited during the phase of sepsis as a feedback effect. Thus, silencing of *Fto* may herald a better treatment outcome.

### Preparation of *Fto* siRNA-Loaded Liposomes

**Figure 2:**
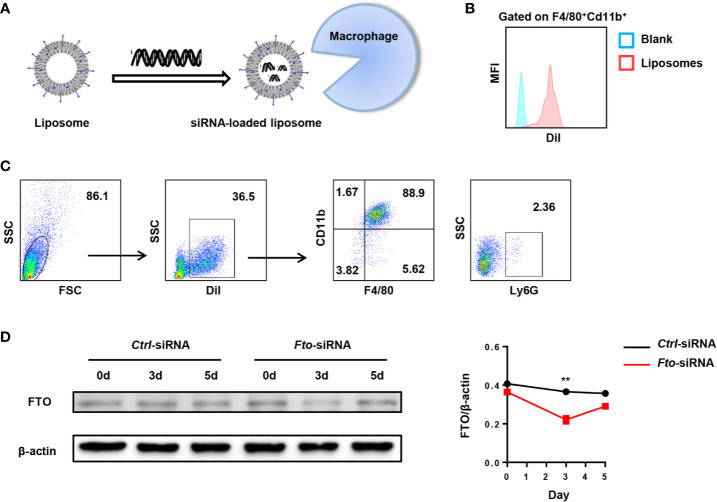
Preparation of *Fto* siRNA-loaded liposomes. DiI-loaded liposomes were intraperitoneally injected in mice and the peritoneal fluid was collected for fluorescence analysis. **(A)** Schematic diagram of the preparation process of *Fto* siRNA-loaded liposomes. **(B)** The mean fluorescence intensity of DiI in F4/80^+^CD11b^+^ macrophages. **(C)** Flow cytometry analysis of the liposomes distribution in the mouse peritoneal fluid. **(D)** Temporal changes in FTO expression in the peritoneal macrophages after *Fto* siRNA-loaded liposomes injection. Data are shown as mean ± SEM; **P < 0.01 (two-tailed unpaired t-test).

For *in vivo* experiments, we designed *Fto* siRNA sequence to inhibit FTO expression in macrophages to verify the hypothesis. Firstly, we examined the biodistribution of *Fto* siRNA-loaded liposomes in C57BL/6 mice (**Figure 2A**). DiI is a lipophilic membrane dye used to label the lipid mixture. The DiI-loaded liposomes were intraperitoneally injected into mice and the peritoneal fluid was collected for fluorescence analysis. Then we measured the fluorescence intensity of DiI in F4/80^+^CD11b^+^ macrophages. The result suggested siRNA-loaded liposomes efficiently targeted macrophages (**Figure 2B**). Next, we detected the DiI^+^ cells by flow cytometry to figure out the cellular localization of liposomes. Surprisingly, the majority of DiI^+^ cells were F4/80^+^CD11b^+^macrophages but not other monocytes, such as dendritic cells and neutrophils (**Figure 2C**). To further clarify the appropriate time interval for the treatment of siRNA-loaded liposomes, we evaluated the FTO expression in the peritoneal macrophages after injection of *Fto* siRNA-loaded liposomes. We found that the expression of FTO decreased significantly on the 3rd day after liposomes administration, while the expression gradually increased on the 5th day (**Figure 2D**). Western blot results showed that the best time interval between administrations was less than 3 days, which suggested us to give injection of liposomes 2 days before LPS treatment. Therefore, the *Fto* siRNA had the highest interference efficiency during the onset of the disease.

In summary, the above results confirmed that liposomes could selectively target peritoneal macrophages, which made them suitable for the treatment of LPS-induced septic shock.

### Intraperitoneal Administration of *Fto* siRNA-Loaded Liposomes Protects Mice Against LPS-Induced Septic Shock

**Figure 3:**
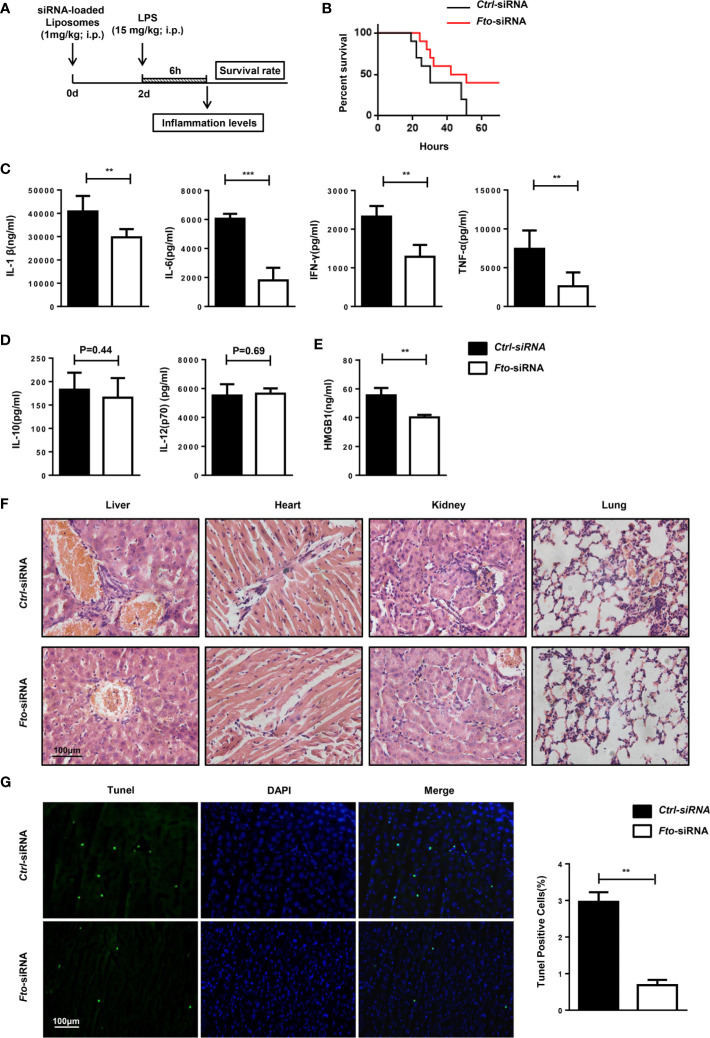
Intraperitoneal administration of *Fto* siRNA liposomes protects mice against LPS-induced septic shock. **(A)** Nanoparticle-mediated *Fto*-siRNA or *ctrl*-siRNA were injected into C57BL/6 mice 2 days before LPS challenge. **(B)** Survival rate of mice injected with siRNA loaded-liposomes (n = 16). **(C, D)** Serum levels of IL-1β, IL-6, TNF-α, IFN-γ, IL-10 and IL-12(p10) were measured 6 h after LPS injection with pretreatment of *Fto*-siRNA or *ctrl*-siRNA liposomes. Plasma cytokine concentrations were measured by ELISA(n = 4 in each group). **(E)** Analysis of serum HMGB1 levels by ELISA. **(F)** Histology of representative tissues stained with hematoxylin and eosin (size bar, 50 μm). **(G)** The TUNEL assay of liver tissue sections. Data are shown as mean ± SEM; **P < 0.01; ***P < 0.001 (two-tailed unpaired t-test).

To evaluate whether FTO could directly participate in the inflammatory response, we tested whether *in vivo* silencing of *Fto* by means of siRNA reduced systemic inflammation and lethal shock in mouse models of sepsis. Nanoparticle-mediated delivery of *Fto* siRNA can silence FTO expression *in vivo*. Then, we injected a lethal dose of LPS intraperitoneally into the mice to induce shock and monitored the lethality rate between the mice pretreated with *scrambled* siRNA (*ctrl*-siRNA) or *Fto*-siRNA before LPS administration (**Figure 3A**).

The mice pretreated with *Fto* siRNA-loaded liposomes showed significantly higher resistance to the lethal effects of LPS in contrast to the ctrl mice that showed 100% lethality within 60 h after 15 mg/kg LPS injection. Although surviving mice pretreated with *Fto* siRNA-loaded liposomes showed shock symptoms at the beginning, they gradually recovered afterwards, indicating a potent protective effect on LPS-induced septic shock (**Figure 3B**). The concentrations of IL-1β, IL-6, IFN-γ and TNF-α in the serum were significantly reduced in mice pretreated with the *Fto* siRNA-loaded liposomes (**Figure 3C**), whereas the amounts of IL-12(p70) and IL-10 remained unchanged (**Figure 3D**). High-mobility group box 1 (HMGB1) was recognized as a late-stage mediator of endotoxin lethality and aggravated the septic shock induced by LPS in mouse models (33, 34). Remarkably, mice treated with *Fto* siRNA-loaded liposomes displayed significantly lower levels of serum HMGB1 (**Figure 3E**). We checked the cytokine intracellular levels in peritoneal macrophages. The results showed *Fto* silencing could reduce the cytokine intracellular levels (**Figure S3**). Consistently, we observed a decrease in the level of immune cell infiltration in the liver, kidney, lung and heart of mice administrated with *Fto* siRNA-loaded liposomes (**Figure 3F**). To examine the protective effect of liposomes on liver damage, tissue sections were treated with TdT and labeled nucleotides and then provided immunofluorescence assay. The TUNEL assay results demonstrated that silencing of *Fto* attenuated LPS induced liver injury and cell apoptosis (**Figure 3G**). Taken together, we could conclude that siRNA-silencing of *Fto* protected mice from LPS-induced endotoxic shock and decreased the inflammatory response *in vivo*.

### Intraperitoneal Administration of Fto-siRNA Liposomes Attenuates Macrophage Activation *in Vivo*

**Figure 4:**
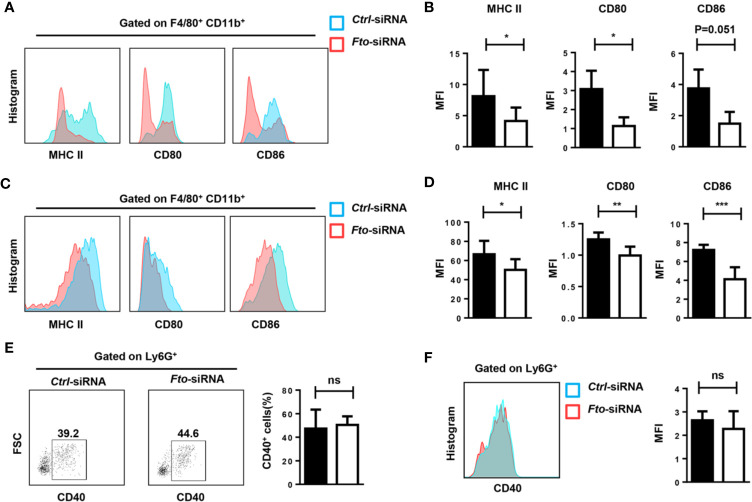
*Fto* knock down attenuates macrophage activation in LPS induced-septic shock. The peritoneal cells **(A, B**, **E)** and splenocytes **(C, D**, **F)** were harvested 6 h after intraperitoneal injection of LPS. **(A–D)** Representative flow cytometry data for analysis of CD80, CD86 and MHC II expression in macrophages, and the mean fluorescence intensity (MFI) values of MHC II,CD80 and CD86 and the expression in F4/80^+^CD11b^+^ macrophages are shown as bar graphs. **(E, F)** Representative flow cytometry data for analysis of CD40 expression in neutrophils, and the mean fluorescence intensity values of CD40 expression in Ly6G^+^ neutrophils are shown in bar graphic figures. Data are shown as mean ± SEM; *P < 0.05; **P < 0.01; ***P < 0.001 (two-tailed unpaired t-test). ns, not significant.

The inflammatory cytokine storm is mainly triggered by macrophages and neutrophils. Therefore, we tested the activation of macrophages and neutrophils in the peritoneum and spleen after LPS stimulation. Administration of *Fto*-siRNA liposomes inhibited MHCII, CD80 and CD86 expression in macrophages (F4/80^+^CD11b^+^) compared to control group (**Figures 4A–D**). However, the activation marker CD40 in Ly6G^+^ neutrophils did not show a significant difference (**Figures 4E, F**). These results suggested that silencing of *Fto* by siRNA attenuates macrophage activation in the peritoneum and spleen, although the neutrophil profiles were not affected.

### The Knocking Down of the *Fto* Gene Expression Inhibits NLRP3 Inflammasome-Mediated IL-1β Secretion Through FoxO1/NF-κB Signaling in Macrophages

**Figure 5:**
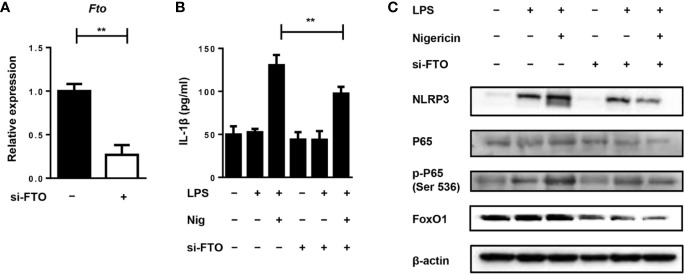
Silencing of Fto inhibits NLRP3 inflammasome-mediated IL-1β secretion through FoxO1/NF-κB signaling pathway in macrophages. **(A)** qPCR results showed treatment of *Fto-*siRNA down-regulated target genes at the mRNA level. **(B)** BMDMs were pre-incubated with *Fto*-siRNA or *ctrl-*siRNA and the NLRP3 inflammasome activation was induced by the treatment of LPS and nigericin. The concentration of IL-1β in culture medium was measured by ELISA. **(C)** Immunoblot analysis of NLRP3, p65, phospho-p65 (Ser 536) and FoxO1 expression in BMDMs stimulated with LPS and nigericin. β-actin was used as a loading control. Data are shown as mean ± SEM; **P < 0.01 (two-tailed unpaired t-test).

To further investigate the role of FTO in IL-1β expression, we used siRNA to treat the primary macrophages for the *in vitro* study. Transfection of primary macrophages with *Fto*-siRNA led to a pronounced reduction in the *Fto* mRNA level (**Figure 5A**). Next, we stimulated siRNA-transfected macrophages with LPS and the NLRP3 inflammasome activator, nigericin, to measure IL-1β secretion (**Figure 5B**). Whereas *scrambled* siRNA-transfected macrophages secreted IL-1β, the production of IL-1β was impaired in *Fto* siRNA-transfected cells (**Figure 5B**).

Since NF-κB signaling plays an important role in the inflammatory response induced by LPS, the effect of NF-κB on primary macrophages was determined by Western blot analysis. We found that LPS stimulation markedly induced the phosphorylation of p65 and pretreatment with *Fto*-siRNA significantly suppressed this process (**Figure 5C**). This indicated that silencing *Fto* could inhibit the activation of NF-κB, thereby regulating the expression of pro-inflammatory genes in macrophages. It was noted that *Foxo1* mRNA is a direct substrate of FTO. FoxO1 signaling through NF-κB was involved in coupling pro-inflammatory cytokine production (24). Thus, we want to assess the inhibitory effect of FTO on FoxO1 expression. As expected, treatment with *Fto* siRNA markedly inhibited FoxO1 expression in primary macrophages (**Figure 5C**). Collectively, our data supported that silencing *Fto* suppressed NLRP3 inflammasome-mediated IL-1β production through FoxO1/NF-κB signaling in macrophages.

### Entacapone Targeting of FTO Demethylase Protects Against LPS-Induced Septic Shock

**Figure 6:**
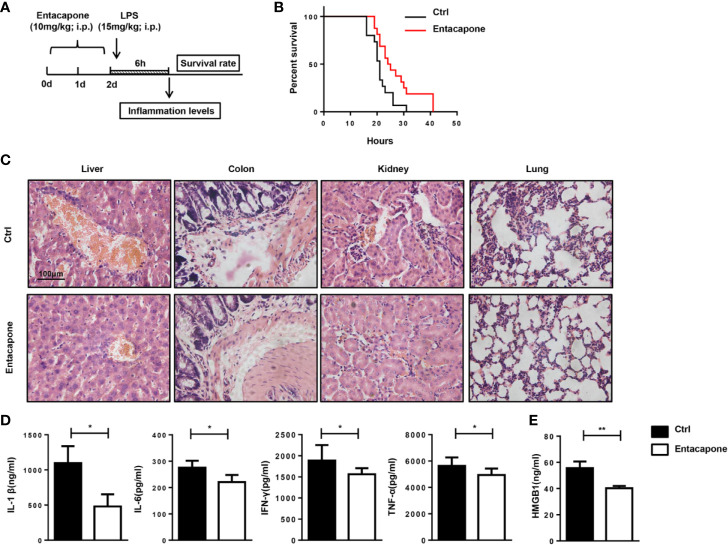
Entacapone targeting of FTO demethylase protects against LPS-induced septic shock *in vivo*. C57BL/6 mice were pre-treated with or without entacapone (10 mg/kg) 2 days prior to LPS injection and 1 h before LPS treatment (15 mg/kg). **(A, B)** Survival rate was monitored continuously (n = 15 in ctrl group; n = 16 in entacapone group). **(C)** Histology of representative tissues stained with hematoxylin and eosin for each experimental group (size bar, 50 μm; n = 4 in each group). **(D)** Serum levels of IL-1β, IL-6, TNF-α, and IFN-γ were measured 6 h after LPS injection with or without entacapone. Plasma cytokine concentrations were measured by ELISA (n = 4 in each group). **(E)** Analysis of serum HMGB1 levels by ELISA. Data are shown as mean ± SEM; *P < 0.05, **P < 0.01. (two-tailed unpaired t-test).

To confirm the protection provided by the blockade of FTO, we tested the therapeutic effect of specific FTO activity inhibitor, entacapone, on the LPS-induced septic shock model. Mice were injected with 10 mg/kg entacapone or the same amount of DMSO before the systemic injection of LPS (**Figure 6A**). Consistent with the results in the *Fto*-siRNA pretreated mice, the mice treated with entacapone before LPS injection showed significantly higher survival rate than the control group (**Figure 6B**). Besides, 20% of entacapone treated mice survived at 36 h after LPS challenge (**Figure 6B**). This result was in sharp contrast with the control group mice that died within 36 hours after LPS administration, and clearly showed that inhibition of FTO had a positive effect on the survival of endotoxin shock. Based on the results, HE staining showed that the immune cell infiltration in the colon, liver, kidney, and lung of the control group mice was more severe than that of the entacapone-pretreated mice (**Figure 6C**). Consistent with this observation, the serum concentrations of IL-1β, IL-6, IFN-γ and TNF-α in mice were significantly reduced by pretreatment with entacapone (**Figure 6D**). The serum HMGB1 level substantially decreased in entacapone treated mice (**Figure 6E**). Thus, these results suggested that FTO inhibition by using the specific inhibitor might be beneficial for septic shock treatment.

### Entacapone Treatment Attenuates Macrophage Activation in LPS-Induced Septic Shock

**Figure 7:**
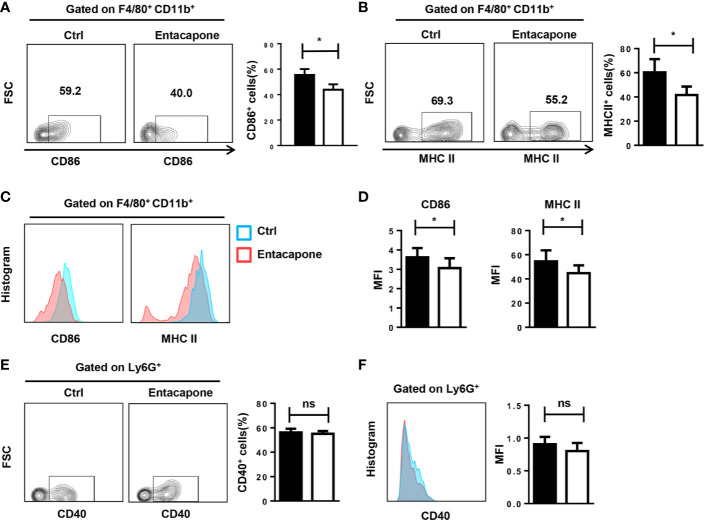
Entacapone treatment attenuates macrophage activation in LPS-induced septic shock. C57BL/6 mice were pre-treated with or without entacapone (10 mg/kg) 2 days prior to LPS injection and 1 h before LPS treatment (15 mg/kg). The peritoneal cells **(A, B**, **E)** and splenocytes **(C, D**, **F)** were harvested 6 h after intraperitoneal injection of LPS. **(A–D)** Representative flow cytometry data for analysis of CD86 and MHC II expression in macrophages, and the mean fluorescence intensity values of CD86 and MHC II expression in F4/80^+^CD11b^+^ macrophages are shown as bar graphs (n=8 for each group). **(E, F)** Representative flow cytometry data for analysis of CD40 expression in neutrophils, and the mean fluorescence intensity values of CD40 expression in Ly6G^+^ neutrophils are shown as a bar graph. Data are shown as mean ± SEM; *P < 0.05 (two-tailed unpaired t-test). ns, not significant.

Next, we tested whether entacapone had an effect on the activation of macrophages and neutrophils in mice treated with LPS. During the inflammatory processes, LPS stimulation significantly increased the expression of CD86 and MHCII in macrophages, and pretreatment with entacapone significantly attenuated LPS-induced macrophage activation in the peritoneum (**Figures 7A, B**) and spleen (**Figures 7C, D**). However, entacapone administration did not affect the neutrophil activation in both ctrl and entacapone treated mice (**Figures 7E, F**).

### Entacapone Inhibits NLRP3 Inflammasome-Mediated IL-1β Secretion

**Figure 8:**
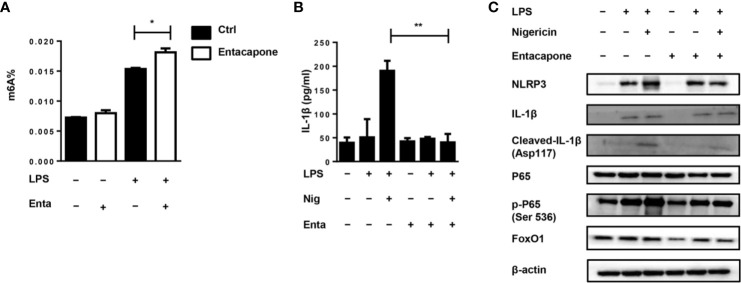
Blockade of FTO inhibits NLRP3 inflammasome-mediated IL-1β secretion. **(A)** BMDMs were pre-incubated with or without entacapone 1 h before LPS stimulation. Then the total RNA was collected after 12 h and the mRNA m^6^A levels were detected by colorimetric ELISA assay. **(B)** BMDMs were pre-incubated with or without entacapone. Then, LPS and nigericin were added to activate the NLRP3 inflammasome. The concentration of IL-1β in culture medium was measured by ELISA. **(C)** Immunoblot analysis of NLRP3, IL-1β, Cleaved-IL-1β (Asp117), FoxO1, P65 and phospho-P65 (Ser 536) expression. β-actin was used as a loading control. Data are shown as mean ± SEM; *P < 0.05; **P < 0.01 (two-tailed unpaired t-test).

It has been found that entacapone could inhibit FTO activity by directly binding to FTO (22). For *in vitro* study, we treated macrophages with entacapone before LPS administration, which significantly increased the m^6^A methylation level compared to the non-treated group (**Figure 8A**). To verify the effect of entacapone on the activation of NLRP3 inflammasomes, we stimulated murine primary macrophages with LPS and inflammasome activator, nigericin, with or without entacapone. The results showed that entacapone significantly reduced secretion of IL-1β induced by nigericin (**Figure 8B**). Consistently, the reduction of NLRP3 and cleaved-IL-1β levels by entacapone treatment was confirmed by immunobloting (**Figure 8C**). Moreover, NF-κB signaling pathway is involved in the process of inflammasome activation. We found that entacapone had a significant inhibitory effect on the phosphorylation of NF-κB enhanced by nigericin treatment. Considering that entacapone elicited its effects on FTO-FoxO1 regulatory axis, we further investigated whether entacapone suppressed the activation of NF-κB signaling *via* the inhibition of FoxO1. Indeed, entacapone dramatically down-regulated the expression of FoxO1 (**Figure 8C**), which was similar to the effect of *Fto*-siRNA treatment. In summary, the results suggested that entacapone inhibited NLRP3 inflammasome-mediated IL-1β secretion through downregulation of FoxO1/NF-κB signaling pathway.

## Discussion

It is worth noting that sepsis is a major clinical problem and the development of novel effective treatments for sepsis is urgently needed. In previous studies, FTO is considered to be associated with the risk of obesity. Recently, FTO has been identified as a m^6^A eraser and plays a critical role in multiple inflammatory disorders. The *Fto* gene has been reported to respond to LPS and to serve as a link between inflammation and metabolic responses. The dominant point mutation of *Fto* gene can reduce fat mass, increase energy expenditure and improve white adipose tissue inflammation (35). Importantly, FTO has been found to be related with the levels of C-reactive protein (36). Greater adiposity conferred by FTO SNPs leads to higher C-reactive protein levels (37). In addition, the present study demonstrated that FTO expression level is higher in the liver of patients with non-alcoholic steatohepatitis (38). Although these reports have shown that FTO is associated with inflammatory disorders, the role of FTO in LPS-induced endotoxin shock remains unknown. In the study, we evaluated the effect of *Fto*-siRNA on the host inflammatory response to LPS-induced endotoxin shock and found that *Fto*-siRNA treated mice showed a higher survival rate compared with control mice. In addition, serum pro-inflammatory cytokines were also significantly reduced in mice pretreated with *Fto*-siRNA, indicating that nanomedicine-based gene therapy can be used as a potential treatment strategy for endotoxin shock.

The production of IL-1β mediated by NLRP3 inflammasomes undergoes a two-step signaling process. First, in the priming phase, the synthesis of pro-IL-1β and NLRP3 depends on the activation of NF-κB. Pathogen-related molecular patterns, such as LPS, are recognized by Toll-like receptors and induce inflammatory response (39). In the secondary signals, NLRP3 inflammasome activation can be triggered by various inducers to promote the mature IL-1β production. Therefore, NLRP3 inflammasome has become an attractive target to reduce inflammation. It has been demonstrated that targeting NLRP3 inflammasomes can alleviate the acute inflammation of LPS-induced endotoxin shock (12). In this study, we evaluated the effect of FTO on macrophages after LPS stimulation and revealed the anti-inflammatory function of *Fto*-siRNA at the LPS priming stage. *Fto*-siRNA reduces IL-1β secretion by inhibiting NF-κB activation and suppressing NLRP3 inflammasome activation. Therefore, the protective effect of targeted FTO inhibition is the combined inhibitory effect of the NF-κB pathway and the activation of NLRP3 inflammasome, and ultimately reduces the production of mature IL-1β.

FoxO1 is a forkhead transcription factor involved in mediating the insulin signaling pathway. The m^6^A sites on *Foxo1* mRNA can be demethylated by FTO to up-regulate FoxO1 expression (22). The FoxO1 pathway regulates multiple cellular processes, such as inflammatory responses, gluconeogenesis, and apoptosis (40–42). It is well established that FoxO1 promotes the production of pro-inflammatory cytokines in insulin resistant hepatocytes (43). FoxO1 has also been confirmed to participate in the regulation of IL-1β production in macrophages (24), suggesting a critical role of FoxO1 signaling in inflammatory process. Moreover, it has been found that FoxO1 can mediate the activation of NLRP3 inflammasome. Inhibition of FoxO1 by using of the molecule inhibitors could block NLRP3 inflammasome assembly and activation (25). Notably, our data demonstrated that silencing *Fto* could inhibit NLRP3 inflammasome activation by down-regulating the expression of FoxO1. However, whether the protective effect of FTO inhibition involves other signaling pathways is currently under investigation.

Entacapone was initially approved by the FDA as a COMT inhibitor for the treatment of Parkinson’s disease (44). Interestingly, it has found that entacapone exhibits its anti-inflammatory properties through anti-oxidation and anti-inflammatory mechanisms in Ang II-induced kidney damage, rather than changes in renal dopaminergic tension induced by COMT inhibition (45). Therefore, elucidating its in-depth anti-inflammatory mechanism is important to explore the role of entacapone in some inflammatory diseases. Recently, entacapone has been identified as a selective inhibitor of FTO activity and can be used as a “tool compound” to study the function of FTO *in vivo* (22). In our study, we demonstrated convincing evidence at entacapone, targeting FTO, could suppress the activation of NLRP3 inflammasome and reduce the release of mature IL-1β in murine primary macrophages, which induced by LPS and the inflammasome activator. Entacapone administration dramatically inhibited macrophage activation, reduced the tissue damage, and delayed the death in a mouse model of LPS-induced septic shock. Therefore, our study provides a new mechanism for the protective effect of entacapone on LPS-induced endotoxic shock and suggests that entacapone may be a promising therapeutic strategy for sepsis in clinical settings.

Our research still has limitations. Since we have confirmed the role of targeted inhibition of FTO in LPS-induce septic shock, we should use conditional knockout mice for further verification. Besides, we have clarified FTO inhibition could suppress NLRP3 inflammasome activation through down-regulating the expression of FoxO1. Therefore, additional experiments should be conducted in animals with FoxO1 overexpression to reversely verify this result.

**Figure 9:**
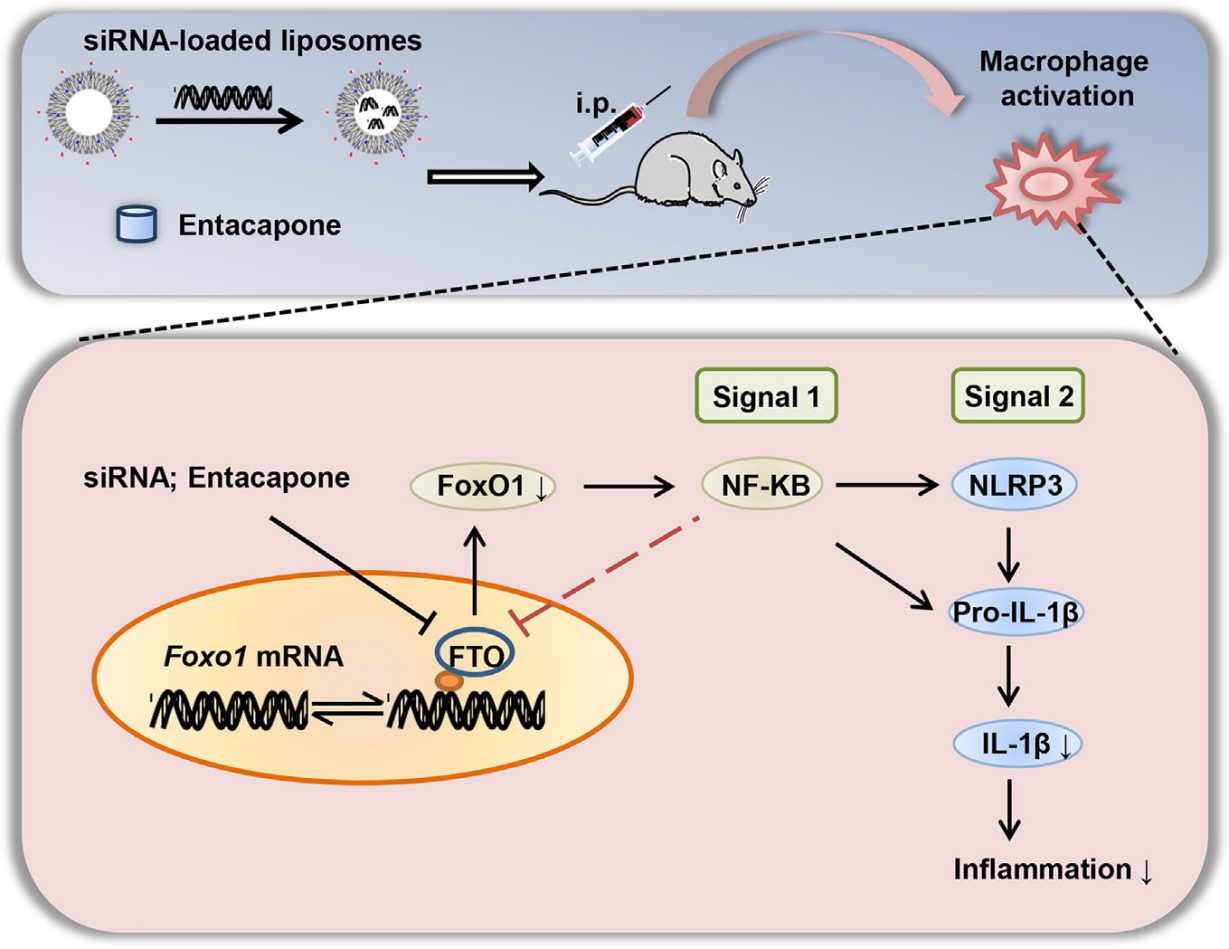
A diagram shows that targeted inhibition of FTO demethylase protects against LPS-induced septic shock by suppressing NLRP3 inflammasome.

In summary, we elucidated that FTO is involved in inflammatory response of LPS-induced septic shock. The nanoparticle-mediated *Fto*-siRNA delivery or entacapone administration dramatically inhibited macrophage activation, reduced the tissue damage, and improved survival in a mouse model of LPS-induced endotoxic shock (**Figure 9**). Mechanistically, inhibition of FTO could inhibit NLRP3 inflammasome through FoxO1/NF-κB signaling in macrophages. Therefore, targeting FTO is promising for the treatment of sepsis.

## Data Availability Statement

The original contributions presented in the study are included in the article/**Supplementary Material**. Further inquiries can be directed to the corresponding authors.

## Ethics Statement

The studies involving human participants were reviewed and approved by Ethics Committee of Tongji Medical College of Huazhong University of Science and Technology. The patients/participants provided their written informed consent to participate in this study. The animal study was reviewed and approved by Tongji Hospital Animal Care and Use Committee.

## Author Contributions

JLu conducted most of the studies and drafted the manuscript. FW and FS contributed to the study design. FW provided help with the flow cytometry analyses. TY, QZ, and CY jointly performed some of the experiments. SR was involved in animal breeding. PY, FX, QY, and SZ contributed to the study design and review of the manuscript. C-YW and JLi designed the research, interpreted the data, and revised the paper. All authors contributed to the article and approved the submitted version.

## Funding

This work was supported by the Ministry of Science and Technology (2016YFC1305002 and 2017YFC1309603), the National Natural Science Foundation of China (81530024, 91749207, 81920108009, 81770823, 81670729, and 81873656), NHC Drug Discovery Program (2017ZX09304022-07), the Integrated Innovative Team for Major Human Diseases Program of Tongji Medical College, Huazhong University of Science and Technology, and the Innovative Funding for Translational Research from Tongji Hospital.

## Conflict of Interest

The authors declare that the research was conducted in the absence of any commercial or financial relationships that could be construed as a potential conflict of interest.
