# Supplementary material for: Targeted Inhibition of FTO Demethylase Protects Mice Against LPS-Induced Septic Shock by Suppressing NLRP3 Inflammasome
Source: Front Immunol. 2021 May 4;12:663295. doi: 10.3389/fimmu.2021.663295 (PMC8128997; doi:10.3389/fimmu.2021.663295)
Supplement: Supplementary file 1 [file DataSheet_1.pdf]

Supplementary Materials

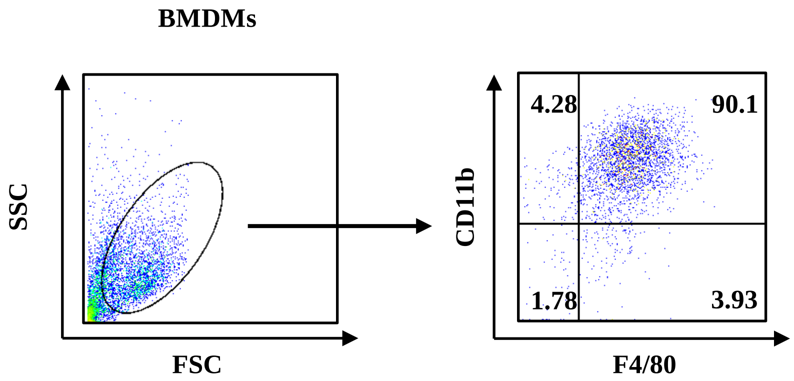

Supplementary Figure 1. Murine bone marrow derived macrophages (BMDMs) were differentiated with macrophage colony stimulating factor for 7 days. Then the differentiated cells were collected for flow cytometry analysis.

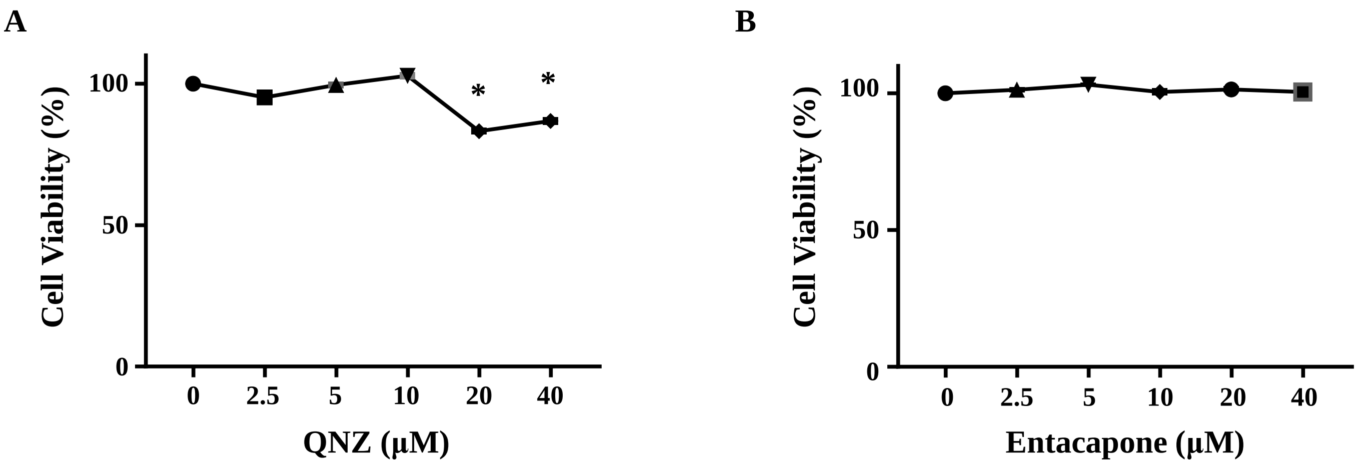

Supplementary Figure 2. The cytotoxic effect of QNZ **(A)** and Entacapone **(B)** treatment on BMDMs for 24 h was determined using a Cell Counting Kit-8 assay. Data are shown as mean ± SEM; *P < 0.05 (two-tailed unpaired t-test).

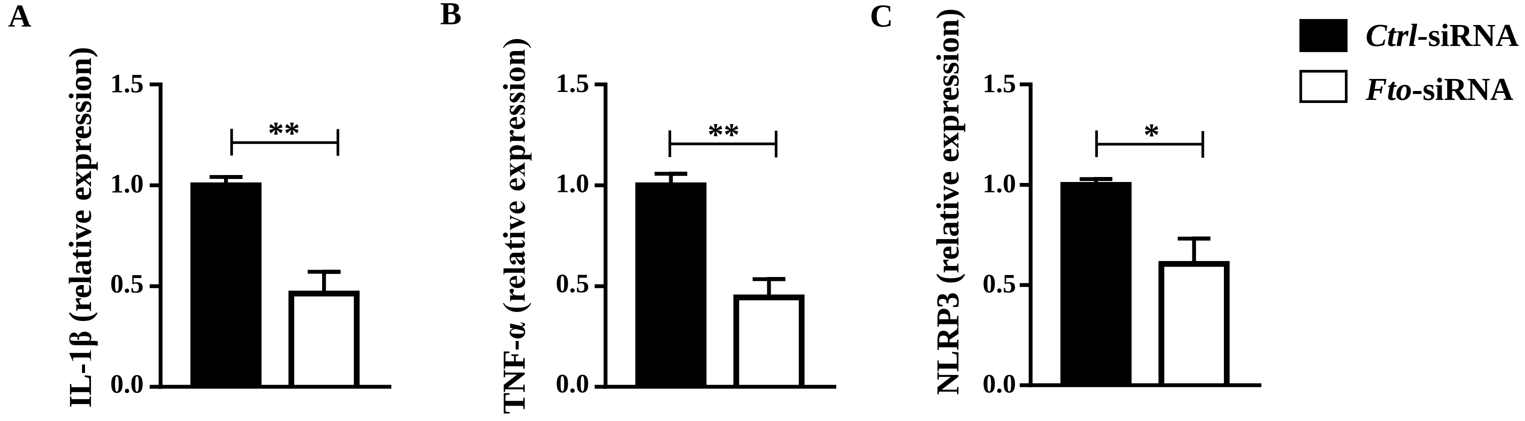

Supplementary Figure 3. Intraperitoneal administration of *Fto* siRNA liposomes inhibits the cytokine intracellular levels in macrophages. Nanoparticle-mediated *Fto*-siRNA or *ctrl*-siRNA were injected into C57BL/6 mice 2 days before LPS challenge. The peritoneal macrophages were harvested 6 h after intraperitoneal injection of LPS. **(A-C)** qPCR analysis of expression of IL-1β, TNF-α and NLRP3 in peritoneal macrophages. Data are shown as mean ± SEM; *P < 0.05; **P < 0.01 (two-tailed unpaired t-test).

Supplementary Table 1. Characteristics of enrolled healthy volunteers and patients with sepsis

	Control (n=24)	Septic patients (n=15)
**Age**, mean ± SD	47.58 ± 14.01	56.4 ± 14.33
**Gender**, N (%)		
Female	4 (16.7%)	0 (0%)
Male	20 (83.3%)	15 (100%)
**Clinical data** (only for septic patients)		
**Associated pathologies**, N (%)		
Tetanus	3 (12.5%)	
Pneumonia	1 (4.17%)	
Hepatitis B cirrhosis	1 (4.17%)	
Intraventricular hemorrhage	2 (8.33%)	
**WBCs** (10^9/L) mean ± SD	10.74 ± 4.18	
**Lymphocytes** (10^9/L) mean ± SD	1.21 ± 0.83	
**SOFA** mean ± SD	9.83 ± 6.59	
**APACHEII** mean ± SD	27.33 ± 12.52	
